# ON and OFF retinal ganglion cells differentially regulate serotonergic and GABAergic activity in the dorsal raphe nucleus

**DOI:** 10.1038/srep26060

**Published:** 2016-05-16

**Authors:** Ting Zhang, Lu Huang, Li Zhang, Minjie Tan, Mingliang Pu, Gary E. Pickard, Kwok-Fai So, Chaoran Ren

**Affiliations:** 1 Guangdong-Hong Kong-Macau Institute of CNS Regeneration, Jinan University, Guangzhou 510632, PR China; 2 Guangdong Medical Key Laboratory of Brain Function and Diseases, Jinan University, Guangzhou 510632, PR China; 3 GHM Collaboration and Innovation Center for Tissue Regeneration and Repair, Jinan University, Guangzhou 510632, PR China; 4 Department of Anatomy, School of Basic Medical Sciences, Peking University, Beijing, 100191 PR China; 5 Key Laboratory on Machine Perception (Ministry of Education), Peking University, Beijing, 100191 PR China; 6 Key Laboratory for Visual Impairment and Restoration (Ministry of Education), Peking University, Beijing, 100191 PR China; 7 School of Veterinary Medicine and Biomedical Sciences, University of Nebraska, Lincoln, NE 68583 USA; 8 Department of Ophthalmology and Visual Sciences, University of Nebraska Medical Center, Omaha, NE 68198 USA; 9 Department of Ophthalmology and State Key Laboratory of Brain and Cognitive Sciences, The University of Hong Kong, Hong Kong, PR China; 10 Co-innovation Center of Neuroregeneration, Nantong University, Jiangsu, PR China

## Abstract

The dorsal raphe nucleus (DRN), the major source of serotonergic input to the forebrain, receives excitatory input from the retina that can modulate serotonin levels and depressive-like behavior. In the Mongolian gerbil, retinal ganglion cells (RGCs) with alpha-like morphological and Y-like physiological properties innervate the DRN with ON DRN-projecting RGCs out numbering OFF DRN-projecting RGCs. The DRN neurons targeted by ON and OFF RGCs are unknown. To explore retino-raphe anatomical organization, retinal afferents labeled with Cholera toxin B were examined for association with the postsynaptic protein PSD-95. Synaptic associations between retinal afferents and DRN serotonergic and GABAergic neurons were observed. To explore retino-raphe functional organization, light-evoked c-fos expression was examined. Light significantly increased the number of DRN serotonergic and GABAergic cells expressing c-Fos. When ON RGCs were rendered silent while enhancing the firing rate of OFF RGCs, c-Fos expression was greatly increased in DRN serotonergic neurons suggesting that OFF DRN-projecting RGCs predominately activate serotonergic neurons whereas ON DRN-projecting RGCs mainly target GABAergic neurons. Direct glutamatergic retinal input to DRN 5-HT neurons contributes to the complex excitatory drive regulating these cells. Light, via the retinoraphe pathway can modify DRN 5-HT neuron activity which may play a role in modulating affective behavior.

The dorsal raphe nucleus (DRN) of the mesencephalon/rostral pons contains the majority of neurons in the brain that use serotonin (5-HT) as a neurotransmitter^1^. Through activation of a diverse assortment of receptors distributed throughout the forebrain (14 5-HT receptors subtypes have been described to date in mammals), 5-HT released from DRN neurons modulates a broad range of physiological functions including learning and memory, reward and punishment, conditioned fear, stress, sleep and circadian rhythms, and affective behavior^2^,^3^,^4^,^5^,^6^,^7^,^8^,^9^,^10^. DRN 5-HT neuronal activity is regulated by the complex interaction between glutamatergic excitatory and GABAergic inhibitory neurotransmission arising from both extra-raphe and local sources^11^,^12^,^13^,^14^,^15^. The glutamatergic afferents to the DRN from the medial prefrontal cortex and lateral habenula begin to illustrate this complexity. These afferents target DRN GABAergic interneurons, which in turn inhibit DRN 5-HT neurons via activation of GABA_A_ receptors^16^,^17^,^18^. These same cortical and brainstem structures also send direct excitatory inputs to DRN 5-HT neurons^14^; determining how these and other afferent signals are integrated by DRN 5-HT neurons remains an ongoing challenge.

In addition to the many glutamatergic afferents from sites in the forebrain and brainstem, the DRN also receives glutamatergic excitatory input from the retina, although details regarding DRN-projecting retinal ganglion cells (RGCs) have only recently begun to emerge^19^. RGC projections to the DRN have been described in several species including primates but have been examined most extensively in the Mongolian gerbil. Of the many types of ganglion cell that have been described in the mammalian retina, in the gerbil more than 85% of the DRN-projecting RGCs have alpha morphologic and Y-like physiologic characteristics^20^. The DRN-projecting alpha/Y-like RGCs are very similar to the classic alpha/Y RGC type described originally in the cat retina (i.e., large soma, transient responses and non-linear spatial summation) and now considered to be a highly conserved RGC type found in species ranging from frogs to primates^21^.

Subpopulations of alpha/Y RGCs have dendrites that stratify in either the proximal or distal inner plexiform layer of the retina corresponding to their ON-center and OFF-center receptive fields (ON RGCs increase firing in response to light increments whereas OFF RGCs increase firing in response to light decrements). OFF alpha/Y RGCs have narrower receptive-field centers and thus to cover the retina OFF cells typically outnumber their ON partners nearly 2-fold^22^. In the gerbil retina, both ON-center and OFF-center alpha/Y-like RGCs project to the DRN but the proportion of ON and OFF cells is strongly biased toward ON cells (≈4 ON: 1 OFF)^20^. The predominance of ON alpha/Y-like RGCs innervating the DRN is therefore unexpected considering the prevalence of OFF alpha/Y RGCs in the retina. It has been suggested that the excess of OFF cells in the retina is related to information available in natural scenes which contain more regions of negative than positive contrast^22^. It would seem that principles that apply to retinal organization regarding image-forming visual circuits may not apply equally to non-image forming retinal circuits that supply information to non-visual targets in the brain such as the DRN. Perhaps the DRN neurons that ON and OFF alpha/Y-like RGCs communicate with (synapse on) is more important in determining their relative proportion in this circuitry than is the contrast information in the natural scene.

Currently the phenotype of DRN neurons innervated by ON and OFF alpha/Y-like RGCs is unknown. Indirect evidence suggests that DRN-projecting OFF alpha/Y-like cells may contribute to the regulation of DRN 5-HT production and/or neurotransmission. Apoptosis of the outer retina can be induced by *N*-methyl-*N*-nitrosourea (MNU), an alkylating agent that results in the rapid elimination of all rod and cone photoreceptors after a single parenteral injection^23^,^24^. In the absence of rods and cones the spontaneous firing rate of all OFF RGCs increases dramatically^25^,^26^; when photoreceptor to ON bipolar synaptic transmission is blocked pharmacologically spontaneous activity in ON RGCs is abolished^27^,^28^. In MNU-treated animals with OFF RGCs firing at a high rate, it was found that DRN serotonin levels were increased while depressive-like behavior was reduced; silencing OFF RGCs or eliminating DRN-projecting RGCs reversed these effects^26^. These findings suggest a potential direct OFF alpha/Y-like RGC projection to DRN 5-HT neurons.

Presently we sought to determine if DRN-projecting RGCs innervate 5-HT and/or GABA neurons. We used intraocular injection of Cholera toxin B subunit (CTB) to label retinal processes in the DRN in conjunction with immunostaining for the glutamatergic receptor postsynaptic scaffold protein PSD-95, combined with immunocytochemical identification of 5-HT and GABA neurons and confocal microscopy. DRN 5-HT and GABA activity was also evaluated using c-Fos expression in animals maintained under different lighting conditions and in the model of MNU-induced photoreceptor apoptosis in which OFF RGC spontaneous firing rate is increased. We report a previously unknown level of complexity in the retinal input to the DRN: the less numerous DRN-projecting OFF Y RGCs primarily activate 5-HT neurons, whereas the more numerous DRN-projecting ON Y RGCs mainly activate GABAergic neurons.

## Materials and Methods

### Animals

Male Mongolian gerbils (*Meriones unguiculatus*) (2–3 months old, 65–77 g) were used. Animals were individually housed and maintained in 12 light: 12 dark (LD) conditions (lights on at 0900 h) with food and water provided *ad libitum*. All procedures were performed in accordance with Jinan University guidelines for animal research and the *Association for Research in Vision and Ophthalmology* Statement for the Use of Animals in Ophthalmic and Vision Research. The experimental animal protocol was approved by Jinan University Institutional Animal Care and Use Committee.

### Light stimulation

Animals were randomly assigned to two groups: 1) a light stimulation group (L), in which the animals were kept in complete darkness overnight from light offset at 2100 h until 0900 h the next day when they were exposed to 90 min of white light (3000 lux) in their home cage; and 2) a dark control group (D) was treated similarly except lights remained off. At 1030 h, all animals were anesthetized (0.25 g/kg, Tribromeoethanol, IP) and perfused transcardially with 0.9% saline followed by 4% paraformaldehyde (PFA) in 0.1 M phosphate-buffered saline (PBS). Brains and eyes were removed and c-Fos immunocytochemistry was performed (see below). c-Fos expression was determined at 90 min after light onset based on the literature and our previous work examining light-induced c-Fos expression in the brain and retina^29^,^30^.

### Specific inhibition of the retinal ON pathway

Specific inhibition of the retinal ON pathway was performed as previously described^29^. Briefly, at approximately 0840 h animals were anesthetized by isofluorane (2.5–5%) inhalation anesthesia. Under dim red light (5 lux), 2 μl of 1 mM L-AP4 (L-(+)-2-amino-4-phosphonobutyric acid; Tocris Bioscience) in sterile 0.9% saline was injected into the vitreous body of both eyes using a glass micropipette attached to a Nanoject II (Drummond Scientific). After recovering from anesthesia (≈10 min), animals were exposed to 90 min of light stimulation and killed at 1030 h as described above.

### Selective elimination of retinal photoreceptors

Selective elimination of rod and cone photoreceptors was performed as previously described^26^. Briefly, animals were anesthetized, and then received an IP injection of MNU (N1517, Sigma-Aldrich) (80 mg/kg) and returned to their home cage maintained under LD conditions for at least 7 days. Some animals were killed 7 days after MNU-treatment and 40 μm cryostat sections were stained with DAPI to document photoreceptor loss.

### Physiological Recording of RGCs

CTB injections into the DRN and recording of DRN-projecting RGCs were previously described^26^. Recorded CTB-labeled RGCs that showed no spontaneous activity (n = 37) in MNU-treated animals were filled and subsequently determined to have dendrites stratifying in the proximal inner plexiform layer of the retina (i.e., ON RGCs). Recorded CTB-labeled RGCs that were spontaneously active (n = 10) in MNU-treated animals were filled and subsequently determined to have dendrites stratifying in the distal inner plexiform layer of the retina (i.e., OFF RGCs).

### Anterograde labeling of axonal terminals of dorsal raphe projecting retinal ganglion cells

Briefly, animals were anesthetized, 0.5% Proparacaine hydrochloride was applied to the cornea, and a 35 g needle attached to a 5.0 μl Hamilton microsyringe was inserted intravitreally at the temporal cornea-conjunctival margin. 2.0 μl of 2% (w/v) CTB-conjugated Alexa Fluor 488 (C-22841, Molecular Probes, Invitrogen) dissolved in 2% dimethyl sulfoxide were slowly injected over 3 min. The needle was held in place for 5 min, withdrawn and the injected site was washed with saline and Bacitracin was applied.

### Immunocytochemistry

All animals were anesthetized (0.25 g/kg, Tribromeoethanol, IP) and perfused intracardially with 0.9% saline followed by 4% paraformaldehyde in phosphate-buffered saline (PBS). Brains and eyes were removed.

Double and triple-labeling was performed on free-floating 40 μm thick cryostat sections incubated in blocking solution for 1 h before primary antibodies were applied. For c-Fos and TPH or GABA double-labeling, were placed in blocking solution for 1 h before incubation in a mixture of primary antibodies against c-Fos (rabbit, 1:30,000; PC38T, Calbiochem) and tryptophan hydroxylase (mouse, TPH; 1:1000; T8575, Sigma-Aldrich), or c-Fos and GABA (mouse, 1:1000; A0310, Sigma-Aldrich) (36 h at 4 °C). Sections were then incubated with corresponding secondary antibodies at a dilution of 1:400 for 6 h at room temperature: goat anti-rabbit Alexa 488(107909, Jackson ImmunoResearch) and goat anti-mouse Alexa 594 (115-587-003, Jackson ImmunoResearch), and cover-slipped in anti-fading aqueous mounting medium (EMS, Hatfield, PA).

For triple-labeling of CTB/TPH/PSD-95 or CTB/GABA/PSD-95, 7 days after CTB intraocular injection animals were perfused with 0.9% saline followed by 4% paraformaldehyde in PBS. After blocking solution a mixture of primary antibodies either against TPH and PSD-95 (rabbit, 1:200, 51-6900, Invitrogen), or GABA and PSD-95 was applied for 36 h (4 °C) and then secondary antibodies at a dilution of 1:300 for 6 h at room temperature: goat anti-mouse Alexa 594 (as above) and donkey anti-Rabbit Alexa 647 (A31573, Life technologies), rinsed in 0.1 M PBS 6X 10 min and cover-slipped (as above).

Retinal whole mounts from a subset of animals used for triple-labeling experiments were immunostained for c-Fos expression. After the animals were anesthetized, the eyes were removed and the retina was dissected from the eyecup, mounted on filter paper, post-fixed 1 h in 4% PFA, removed from the filter paper, washed in 0.1 M PBS 3X 10 min, incubated in CAS-Block^TM^ (1673905A, Life technologies) containing 0.3% Triton-X-100 for 1 h, incubated in c-Fos antibody (as above) for 48 h at 4 °C followed by 3X PBS before incubation with Dylight 488 goat-anti-rabbit IgG (Vector Laboratories) at 1:400 for 6 h at room temperature.

The method for immunocytochemical staining of cholinergic amacrine cells in the retina was performed as previously described^31^. Briefly, retinas were fixed for 1 hour in 4% paraformaldehyde in 0.1 M PBS followed by rinsing in 0.1 M PBS (3X 10 min) and placed in 10% normal goat serum containing 2% Trition-X-100 for one hour at room temperature. Retinas were then incubated in goat-anti-ChAT antibody (1:200, AB144P, Millipore) for 48 hours at 4 °C. This was followed by 6 times rinsing in 0.1 M PBS (6X 10 min) and then incubation with a secondary antibody Alexa Fluor 594 donkey anti-goat IgG (1:400, A-11058, Molecular Probes) for 6 hours at room temperature. Finally, all retinas were rinsed in 0.1 M PBS and cover-slipped (as above).

### Confocal microscopy and three-dimensional reconstruction

Images were collected with a confocal laser-scanning microscope (Zeiss, LSM700). For triple-labeling the Z-axis interval was set at 0.3 μm and areas of interest were scanned with a 63X oil immersion objective, and zoomed 4 times by digital magnification. A montage of optical section stacks was created, projected to a 0° X–Y plane and a 90° X–Z plane to obtain a 3-D reconstruction. Using Image J and Photoshop CS5 (Adobe Corp., San Jose, California, USA), contrast and brightness were adjusted and images were pseudo-colored.

### Statistical analysis

For quantification of c-Fos+ cells and c-Fos+ GABA+ or TPH+ cells, 4 serial sections through the caudal DRND/L were examined/animal and analyzed using a one-way ANOVA. Data are expressed as the mean ± SEM. Statistical significance was set at p < 0.05.

## Results

### Retinal ganglion cells form synaptic connections with GABA and serotonin neurons in the DRN

**Figure 1:**
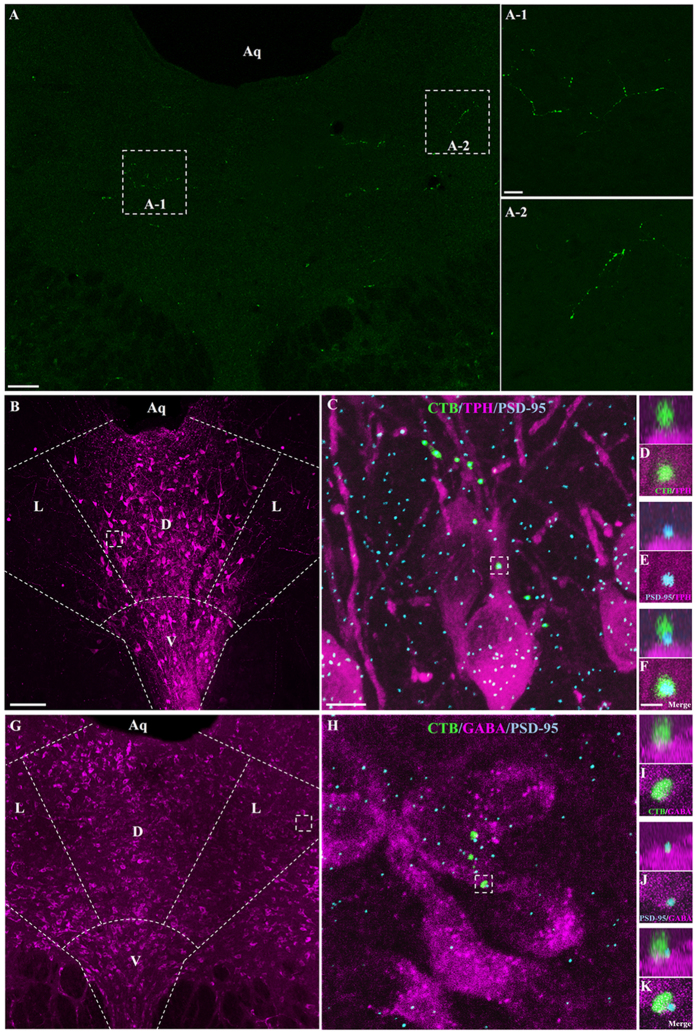
DRN-projecting RGCs contact 5-HT and GABA neurons in the DRND/L. (**A**) CTB-labeled RGC processes in DRN. (**B**) Z-stack image of TPH neurons (magenta) in DRND/L. Dorsal raphe nucleus, lateral: L; dorsal raphe nucleus, dorsal: D; dorsal raphe nucleus, ventral: V. (**C**) Box in (**B**). Note a CTB-labeled RGC process associated with TPH neurons (Green: CTB; Blue: PSD-95). (**D**–**F**) Box in C. (**D**–**F**) Note that a CTB+ bouton is in apposition to a PSD-95 puncta on a TPH+ neuron. (**G**) Z-stack image of GABA neurons (magenta) in DRND/L. (**H**) Box in (**G**). Note a CTB-labeled RGC process associated with GABA neurons (Green: CTB; Blue: PSD-95). (**I**–**K**) Box in H. (**I**–**K**) Note that a CTB+ bouton is in apposition to PSD-95 puncta on a GABA+ neuron. Aq: aqueduct. Scale bars: 100 μm in A; 20 μm in A-1 (applies to A-2); 100 μm in B (applies to G); 2 μm in C (applies to H); 1 μm in F (applies to D, E and I to K).

We examined first whether axonal varicosities of DRN-projecting RGCs formed synaptic connections with DRN 5-HT and/or GABA neurons. Following intraocular injection of CTB-Alexa Fluor 488, labeled axonal processes were found distributed mainly in the dorsal and lateral subdivisions of the caudal DRN (DRND/L)^32^ (Fig. 1A). Only a relatively small number of CTB-labeled retinal fibers were observed in individual tissue sections through the DRND/L. However, it is very likely that the density of retinal fibers in the DRN is under represented by CTB-Alexa Fluor 488 anterograde tracing^33^. Tissue sections of the DRND/L with CTB-labeled processes were subjected to immunocytochemical labeling for tryptophan hydroxylase (TPH), the rate limiting enzyme in 5-HT synthesis, or GABA, together with the postsynaptic protein PSD-95, a scaffolding protein associated with glutamatergic receptors. CTB-labeled varicosities of DRN-projecting RGCs were noted to form synaptic connections with 5-HT and GABA neurons (Fig. 1B–K). Varicosities of the glutamatergic CTB-labeled retinal processes abutted the postsynaptic PSD-95 protein and contacts were confirmed in rotated and magnified images (Fig. 1D–F,I–K) suggesting strongly that DRN-projecting RGCs form synaptic contacts with 5-HT and GABA neurons in the DRND/L.

### Light stimulation induces c-Fos expression in the DRN

**Figure 2:**
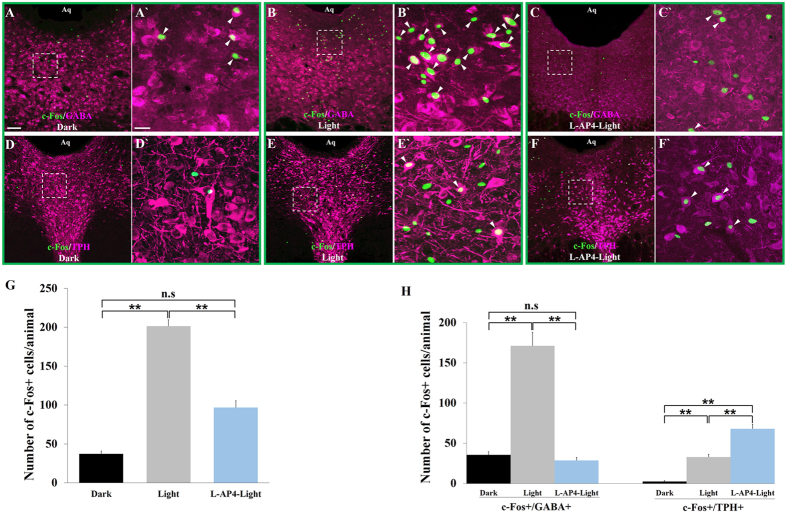
Effect of light on c-Fos expression in DRND/L 5-HT and GABA cells. (**A**–**C**) c-Fos in GABA+ neurons under dark conditions (**A**), after light stimulation (**B**) and in L-AP4 treated animals after light stimulation (**C**) (magenta: GABA; green: c-Fos). A’ is box in A. B’ is box in B. C’ is box in C. (**D**–**F**) c-Fos in TPH+ neurons under dark conditions (**D**), after light stimulation (**E**) and in L-AP4 treated animals after light stimulation (**F**) (magenta: TPH; green: c-Fos). D’ is box in D. E’ is box in E. F’ is box in F. Arrowheads indicate cells co-labeled with c-Fos and GABA or TPH. (**G**) Quantification of c-Fos+ cells in D, L and L-AP4-L groups, n = 5/group. (**H**) Quantification of c-Fos + /GABA+ and c-Fos + /TPH+ cells in D, L and L-AP4-L groups, n = 5/group (**p < 0.001). Scale bars: 100 μm in A (applies to B, C, D, E and F); 20 μm in A’ (applies to B’, C’, D’, E’ and F’).

After establishing that DRN-projecting RGCs were in direct contact with 5-HT and GABA neurons in the DRND/L using morphological procedures, we sought to determine whether RGC input to the DRN regulates neuron activity using light-induced c-Fos expression as a functional readout. Two groups of animals (n = 10/group) were used: animals in the light stimulated group (L) were sacrificed 90 minutes after light onset whereas the control group (D) remained in the dark and was sacrificed at the same time of the day; the DRND/L in both groups was examined for c-Fos expression. The number of c-Fos positive (+) neurons/animal in the DRND/L in the L group was significantly greater than the number of c-Fos positive neurons in the D group (37.4 ± 3.6 vs 201.6 ± 8.1; n = 10/group; p < 0.0001); light stimulation (3000 lux) produced a >5-fold increase in c-Fos+ expressing neurons in the DRND/L (Fig. 2G).

To investigate whether 5-HT and/or GABA DRND/L neurons expressed c-Fos in response to light exposure, five animals from each D and L group were randomly selected and further examined for c-Fos and GABA co-localization and the remaining 5 animals in each group were analyzed for c-Fos and TPH co-localization. A vast majority (87%) of the c-Fos+ cells in the D group were either GABA+ or TPH+: 79.2 ± 2.8% of c-Fos+ cells were GABA+ neurons and 7.8 ± 2.2% of c-Fos+ cells were TPH+ neurons (Fig. 2A,D,H and Table 1). Light exposure increased the number of c-Fos+/GABA+ cells ≈5-fold compared to the D group (35.6 ± 4.1 vs 171.2 ± 16.9, n = 5/group; p < 0.001), whereas the number of c-Fos + /TPH+ cells increased ≈13-fold following light stimulation (2.6 ± 0.9 vs 33.0 ± 3.2, n = 5; p  <  0.001) (Fig. 2B,E,H and Table 1). Thus, light exposure significantly increased the number of GABA+ and TPH+ DRND/L cells expressing c-Fos compared to animals in the dark. Although light had a disproportionately greater effect on 5-HT neurons (13-fold increase vs 5-fold increase in GABA cells, Fig. 2H), following light stimulation the number of c-Fos + /GABA+ cells (171.2 ± 16.9) was significantly greater than the number of c-Fos + /TPH+ cells (33.0 ± 3.2) (Table 1). This could mean that GABA cells receive a greater number of RGC inputs than TPH cells and/or that activated GABA cells inhibit TPH cells. Since DRN-projecting ON Y-like RGCs outnumber DRN-projecting OFF Y-like RGCs approximately 4-fold, ON DRN-projecting RGCs may preferentially innervate DRN GABA neurons. To begin to break down the contributions of ON and OFF DRN-projecting RGCs in the regulation of neurons in the DRND/L, additional experiments were conducted.

### ON and OFF retinal pathways differentially regulate DRN GABA and serotonin neurons

Two different types of experiments were performed: the first examined light-induced Fos expression in the DRN following acute pharmacological suppression of the retinal ON pathway whereas the second examined Fos expression in the DRN after elimination of rods and cones resulting in chronic silencing of ON RGCs while concurrently increasing the spontaneous activity of OFF RGCs.

#### L-AP4 blockade of the retinal ON pathway

It is well documented that L-AP4, an agonist of the mGluR6 receptor blocks transmission of visual signals between photoreceptors and ON-bipolar cells thus blocking input to ON RGCs^34^. To examine the role of OFF RGC input to the DRN in the absence of ON RGC input, 10 animals were intraocularly treated with 1 mM L-AP4 and light-induced c-Fos expression was examined in the DRN. L-AP4 application significantly decreased the total number of light induced c-Fos+ cells in the DRND/L compared to untreated light-stimulated control animals (97.0 ± 8.6 vs 201.6 ± 8.1; p < 0.001) and this was due to the number c-Fos + /GABA+ being reduced to levels comparable to control animals in the dark (35.6 ± 4.1vs 28.8 ± 3.6). Conversely, the number of c-Fos + /TPH+ cells in the DRND/L was significantly increased in L-AP4 treated animals compared to untreated light-stimulated control animals (33.0 ± 3.2 vs 68.0 ± 5.6, p < 0.001) (Fig. 2H and Table 1). These results are consistent with the interpretation that DRN-projecting alpha/Y-like RGCs innervate both GABA and 5-HT cells in the DRND/L but that ON DRN-projecting RGCs predominately innervate GABAergic neurons.

#### MNU-induced rod and cone apoptosis

**Figure 3:**
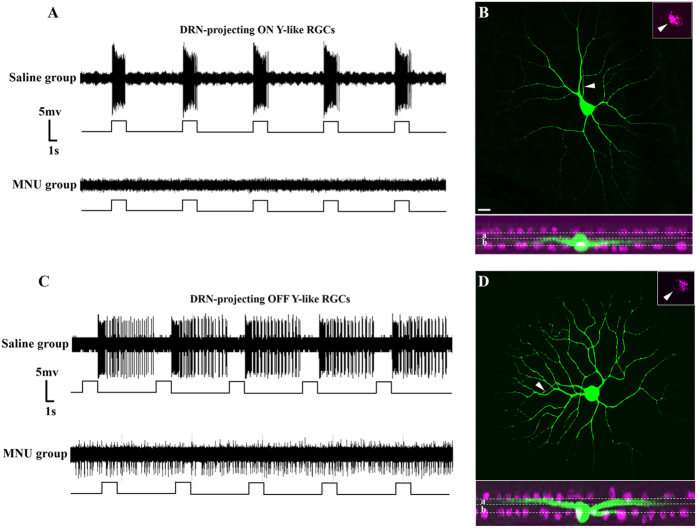
Effect of MNU on the activity of DRN-projecting RGCs. (**A**) Firing patterns of DRN-projecting ON Y-like RGCs in response to visual stimulation. Top trace: Transient discharge pattern of retrogradely labeled ON Y-like RGC (firing increases at light onset) in a saline treated animal. Bottom trace: Loss of light-evoked firing of a retrogradely labeled ON Y-like RGC in an MNU-treated animal (representative of 37 recorded ON cells. (**B**) Dendritic morphology of the recorded DRN-projecting ON Y-like RGC in bottom trace. Arrow in inset at upper right corner points to the CTB retrogradely labeled soma. White dotted lines in lower plane indicated borders of sublamina *a* and *b* of IPL. Cholinergic amacrine cells were labeled and colored magenta. (**C**) Firing patterns of DRN-projecting OFF Y-like RGCs in response to visual stimulation. Top trace: Transient discharge pattern of retrogradely labeled OFF Y-like RGC (firing increases at light offset) in a saline treated animal. Bottom trace: Loss of visual responsiveness and vigorous spontaneous firing of a retorgradely labeled OFF Y-like RGC in an MNU treated animal (representative of 10 recorded OFF cells). (**D**) Dendritic morphology of the recorded DRN-projecting OFF Y-like RGC. Arrow in inset at upper right corner points to the CTB retrogradely labeled soma. White dotted lines in lower plane indicated borders of sublamina *a* and *b* of IPL. Cholinergic amacrine cells were labeled and colored magenta. Scale bar: 20 μm in B, applies to D.

Abolishing the maintained firing activity in the ON pathway following acute L-AP4 application may have also acutely increased the spontaneous firing rate of OFF RGCs; L-AP4 has been reported to increase OFF alpha cell firing rate ≈2-fold in an *in vitro* preparation^27^,^28^. To investigate further the role of ON and OFF DRN-projecting RGCs in the regulation of neural activity in the DRND/L in a model in which RGC activity is chronically altered, 20 animals received a single parenteral injection of MNU, which could result in the rapid and specific ablation of rod and cone photoreceptors in the outer retina^26^. Ablation of rods and cones results in silencing of ON RGCs and chronic activation of DRN-projecting OFF RGCs (Fig. 3)^26^; 16 animals injected with a similar volume of saline served as controls. Saline and MNU-treated animals were examined for c-Fos and GABA or TPH co-localization in the dark and after light stimulation.

**Figure 4:**
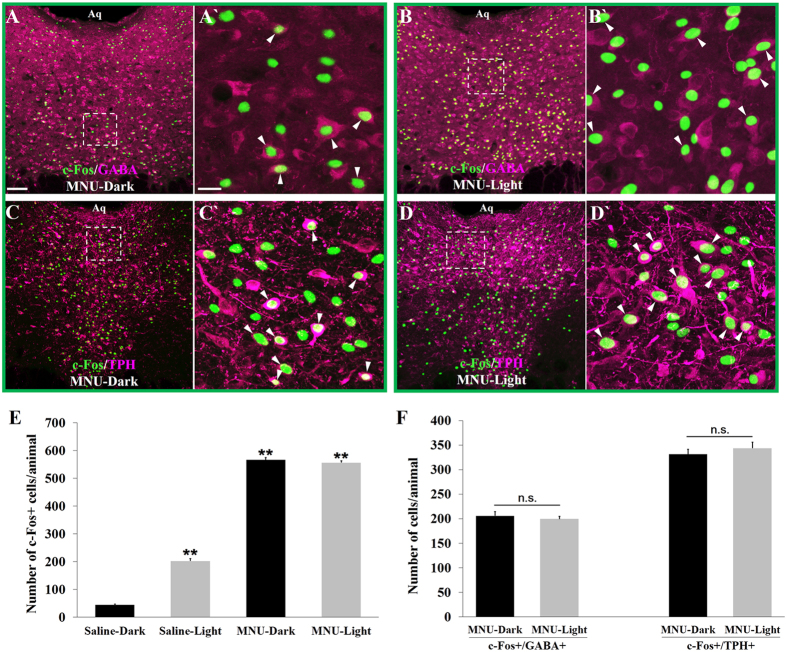
Effect of selective activation of OFF DRN-projecting RGCs on c-Fos in GABA and 5-HT neurons in the DRND/L. (**A**,**B**) c-Fos in GABA+ neurons in MNU-treated animals: in the dark (**A**) and after light stimulation (**B**) (magenta: GABA; green: c-Fos). A’ is box in A. B’ is box in B. (**C**,**D**) c-Fos in TPH+ neurons in MNU-treated animals: in the dark (**C**) and after light stimulation (**D**) (magenta: TPH; green: c-Fos). C’ is box in C. D’ is box in D. Arrowheads indicate cells co-labeled with c-Fos and GABA or TPH. (**E**) Quantification of c-Fos+ cells; n = 8/saline group; n = 10/MNU group (**p < 0.001). (**F**) Quantification of c-Fos + /GABA+ and c-Fos + /TPH+ cells in MNU-D and MNU-L groups (n.s. = not significant). Scale bars: 100 μm in A (applies to B, C and D); 20 μm in A’ (applies to B’, C’ and D’).

The number of c-Fos+ cells in the DRND/L of MNU-D group was significantly increased (≈13-fold) over the number of c-Fos+ cells in saline-D control group (44.8 ± 2.6 vs 566.5 ± 8.3, p < 0.001), and the number of c-Fos+ cells in MNU-L group was significantly increased (≈3 fold) over the number of c-Fos+ cells in saline-L group (201.2 ± 8.2 vs 556.6 ± 6.8, p < 0.001) (Fig. 4E; Table 1). The expression of c-Fos in the DRND/L was indistinguishable between the MNU-L (556.6 ± 6.8) and MNU-D (566.5 ± 8.3) groups (Fig. 4E; Table 1). Thus, MNU-treatment evoked greater DRND/L c-Fos expression than did light stimulation. Since MNU-treatment renders DRN-projecting ON RGCs silent (Fig. 3) while increasing the spontaneous firing rate of DRN-projecting OFF RGCs approximately 6-fold^26^, the increase in DRND/L c-Fos expression in MNU-treated animals is attributed to the increased spontaneous activity of DRN-projecting OFF RGCs.

Immunocytochemical analyses of c-Fos and TPH or GABA co-labeling revealed that MNU treatment increased the number of c-Fos + /GABA+ cells only slightly over saline-L values (saline-L: 165.7 ± 7.8 vs MNU-L: 199.6 ± 5.3), whereas the number of DRND/L c-Fos + /TPH+ neurons was increased 10-fold (saline-L: 34.7 ± 3.8 vs MNU-L: 343.6 ± 12.5) (Fig. 4; Table 1). Since MNU-treatment specifically increases the firing rate of OFF DRN-projecting RGCs, these RGCs appear to preferentially innervate DRN 5-HT neurons. Thus, in MNU-D or MNU-L animals, DRND/L c-Fos + /TPH+ cells outnumbered light activated DRN c-Fos + /GABA+ cells almost two-to-one (Table 1). The opposite is the case in the light exposed animals with intact retinas (saline-L), where DRND/L c-Fos + /GABA+ cells outnumber c-Fos + /TPH+ cells five-to-one (Table 1).

**Figure 5:**
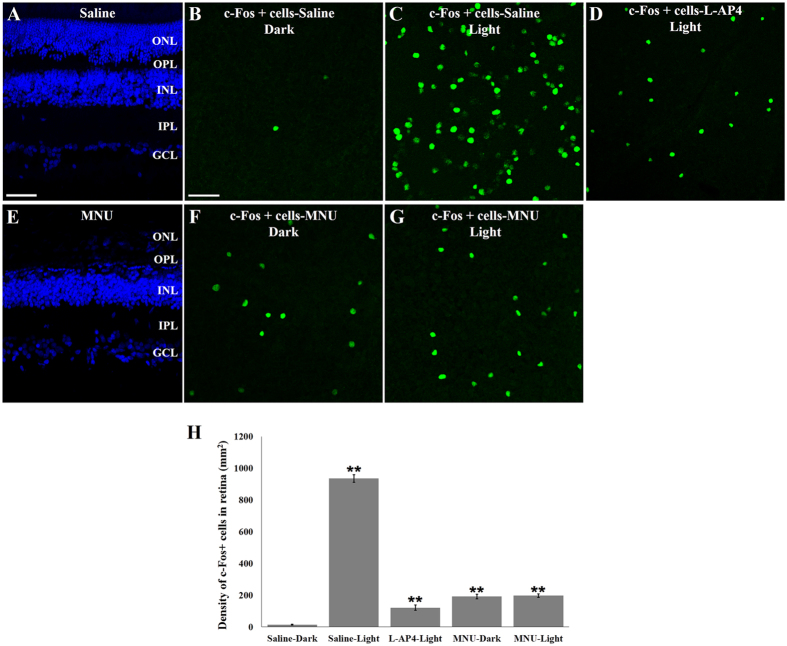
Effect of MNU on outer retina and c-Fos expression in ganglion cell layer. (**A**) Normal retina of Saline-treated animal and (**E**) retina lacking outer nuclear layer (ONL) in MNU-treated animal. (**B**–**D**) c-Fos+ cells in the ganglion cell layer (GCL) of Saline-treated animals in the dark (**B**), after light stimulation (**C**) and in L-AP4 treated animals after light stimulation (**D**). (**F–G**) c-Fos+ cells in the GCL of MNU-treated animals in the dark (**F**) and after light stimulation (**G**). (**H**) Quantification of c-Fos+ cells; n = 5 retinas/group; (*p < 0.05; **p < 0.001) vs Saline-Dark group. Scale bars: 50 μm in A (applies to E); 20 μm in B (applies to C, D, F and G).

The retinas of the L-AP4-treated animals, saline control animals and MNU-treated animals lacking photoreceptors in the outer retina (Fig. 5A,E) were also examined for c-Fos expression in the ganglion cell layer. In retinal whole mounts there were more c-Fos+ cells in the saline-L, L-AP4-L, MNU-D and MNU-L compared to the saline-D controls as expected (Fig. 5). There was also no difference in the number of c-Fos+ cells in the retinas of MNU-D and MNU-L animals consistent with the absence of a response to light due to the ablation of outer retina photoreceptors (Fig. 4H and Table 1). In addition, compared with saline-L group, the number of c-Fos+ cells in the retinas of MNU-treated animals was significantly decreased (Fig. 5H and Table 1). The reduced expression of c-Fos in the retinas of MNU-treated animals is due to the fact that MNU treatment renders all ON RGCs silent while activating OFF RGCs (Fig. 3) whereas light stimulation activates both ON and OFF RGCs.

**Figure 6:**
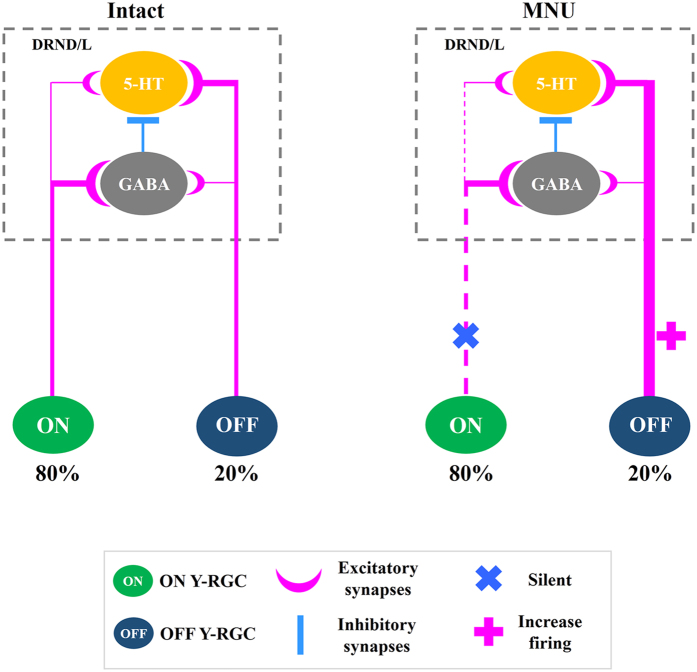
Model of DRN-projecting RGCs to GABA and 5-HT neurons in the DRND/L. ON and OFF Y-like RGCs provide excitatory input to both 5-HT and GABA neurons in the DRND/L with ON RGCs predominately innervating GABA cells and OFF RGCs predominately innervating 5-HT cells. GABA interneurons inhibit 5-HT cells. In the intact animal, light stimulation has a greater quantitative effect of GABA cells due to the 4:1 ratio of ON to OFF DRN-projecting Y RGCs although 5-HT cells. In the MNU-treated animal, ON RGCs fall silent and OFF RGC firing is enhanced resulting in a greater quantitative effect on 5-HT activity (c-Fos + /5-HT cells are 10 fold greater in MNU-treated animal vs light stimulated intact animal) and this effect is independent of the environmental light status due to absence of rod and cone photoreceptors.

The anatomical and functional data described above taken together with our previous work^20^,^26^ and the known role of DRN GABAergic interneurons suggest that most ON DRN-projecting RGCs innervate DRND/L GABA neurons which in turn innervate serotonin neurons. Most OFF DRN-projecting RGCs innervate DRND/L serotonin neurons directly (Fig. 6).

## Discussion

In a previous study we demonstrated for the first time that retinal input to the DRN can modulate 5-HT levels and depressive-like behavior^26^. These observations prompted us to investigate the postsynaptic targets of retinal afferents in the DRN. In the present set of experiments we demonstrated that CTB-labeled RGC presynaptic terminals are in close (synaptic) apposition with the postsynaptic protein PSD-95 (which is associated with glutamatergic receptors) on both 5-HT and GABA neurons in the DRND/L. In addition, we observed that light stimulation evoked increases in c-Fos expression in the DRND/L almost exclusively in 5-HT and GABA neurons; more than 97% of Fos+ cells were GABA or 5-HT neurons. We also report robust c-Fos expression in DRND/L 5-HT neurons in MNU-treated animals suggesting that the population of OFF DRN-projecting RGCs preferentially innervates 5-HT neurons whereas the majority of ON DRN-projecting RGCs (ON DRN-projecting RGCs outnumber OFF DRN-projecting RGCs cells 4:1) preferentially innervates GABA neurons. It is also likely that DRND/L GABA neurons receiving direct input from ON (and OFF) RGCs inhibit DRND/L 5-HT neurons. The anatomical and functional data presented herein taken together with our previous work suggest that RGCs differentially innervate DRND/L 5-HT and GABA neurons thereby adding to the complex glutamatergic regulation of DRN 5-HT activity.

The primary excitatory drive of DRN 5-HT neurons comes from a wide range of glutamatergic inputs from cortical and subcortical regions^12^ and as demonstrated in this study, the retina. The origins of glutamatergic afferents to the DRN can be distinguished by the presence of the specific type of vesicular glutamate transporter used to load glutamate into synaptic vesicles. Axons from subcortical structures and the retina express the VGLUT2 isoform^35^,^36^,^37^,^38^,^39^,^40^ and among axons in the DRN expressing VGLUT1, VGLUT2 or VGLUT3, those expressing VGLUT2 are found most often in close apposition to PSD-95 on 5-HT and non-5-HT neurons^41^. Thus, our data are consistent with previous work indicating that glutamatergic axons expressing VGLUT2 provide excitatory synaptic input to 5-HT and non-5-HT (e.g., GABA) neurons in the DRN.

In the current study, evidence is provided that the retina projects directly to 5-HT and GABA neurons in the DRN. Although the gerbil retinoraphe projection is robust compared to that described for other species the number of CTB-labeled retinal processes observed in the DRN is relatively low^19^. It is very likely however, that retinal processes in the DRN are under-represented when CTB is used as an anterograde tracer. For example, labeled retinal fibers were not reported in the DRN following intraocular CTB injection in the cat whereas a retinoraphe projection was unambiguously described in the cat using intraocular injection of ^3^H-amino acids and by retrograde labeling of RGCs after horseradish peroxidase was injected into the DRN^42^,^43^. We have also recently reported that retrograde labeling of DRN-projecting RGCs reveals more cells than predicted from CTB anterograde labeling of retinal fibers in the DRN^44^. When comparing retinal afferent fiber labeling in the rat DRN using different tracers, it was reported that CTB was a less sensitive tracer than CTB conjugated to HRP^33^. Since retinal fibers are likely under-reported following intraocular CTB application, no attempt was made to quantify retinal synapses on neurons in the DRN. High-resolution neuroanatomical techniques could be employed to quantify retinal synapses in the DRN^12^ if the apparent under representation of the retinoraphe projection can be overcome using more sensitive approaches.

5-HT and GABA neurons in the DRND/L were activated by light. However, it is well documented that GABA neurons predominate in the DRND/L and play a pivotal role in the modulation of serotonergic tone through direct synaptic connections with 5-HT neurons^45^,^46^,^47^. Thus, in our simple model presented in Fig. 6, we postulate that DRND/L 5-HT neurons may receive indirect inhibitory retinal input via GABAergic interneurons and direct excitatory retinal input, similar to the indirect inhibitory input and direct excitatory inputs to the DRN from the medial prefrontal cortex and lateral habenula^14^. In other studies examining c-Fos expression in the DRN following diverse non-light related stimulation (e.g., REM sleep, swim stress, depression), c-Fos expression was observed almost exclusively in DRN GABA neurons, which could then modulate serotonergic tone through inhibitory synaptic connections with 5-HT neurons^48^,^49^,^50^,^51^. The number of GABA and 5-HT cells expressing c-Fos suggests that ON and OFF DRN-projecting RGCs project to both GABA and 5-HT neurons but that ON RGCs primarily innervate GABA neurons and OFF RGCs primarily innervate 5-HT neurons in the DRND/L (Table 1). It is possible that ON and OFF RGCs might even target the same DRN cells. The specific synaptic architecture between glutamatergic retinal afferents and their postsynaptic targets, such as the presynaptic gating of glutamatergic inputs by GABA in the DRN^13^, remains to be determined.

OFF RGCs increase their spontaneous firing rate when synaptic input via rods and cones is severely reduced or eliminated following degeneration of rods and cones^25^, pharmacological blockade^27^,^28^ or after MNU-induced apoptosis of the outer retina^26^. MNU has been shown to specifically target rod and cone photoreceptors in several species causing rapid apoptosis while sparing RGCs^23^,^24^,^26^,^52^ (Fig. 5). In MNU-treated animals, acute silencing of OFF RGC firing reverses the effects on depressive-like behavior^26^. Thus, c-Fos expression observed in the DRN in MNU-treated animals is attributed to increased activity of OFF DRN-projecting RGCs rather than an indirect effect of MNU treatment.

ON DRN-projecting Y RGCs dominate the retinoraphe projection even though OFF Y cells out number ON Y cells 2:1. The reason for this disparity in ON and OFF DRN-projecting RGCs is unknown. OFF RGCs cells are more numerous in the retina because natural images contain more dark information and thus the retina apparently devotes more resources to processing dark contrasts^22^. Since the retinoraphe projection is not a ‘visual’ pathway concerned with visual information, the retinoraphe projection may not need to follow the same pattern as classic retinal circuits. On the other hand, the ON and OFF RGC projection to the DRN shares some features with the visual projections to the thalamus. ON and OFF pathways that begin in the retina maintain their segregation when they innervate neurons in the dorsal lateral geniculate nucleus (dLGN)^53^. The postsynaptic targets of ON and OFF RGCs in the dLGN are glutamatergic relay neurons and GABAergic interneurons^54^. ON RGCs innervate dLGN GABAergic interneurons that synapse on dLGN relay neurons that receive direct OFF RGC input^55^.

DRN serotonergic neurons project in a topographic manner to widespread targets throughout the forebrain and lower brainstem. Serotonergic neurons in the DRND/L project to several structures of the central visual system including the dLGN, superior colliculus, and primary visual cortex where serotonin actively shapes the responses property of visual system networks^19^. In addition, serotonergic neurons in the DRND/L appear to exert inhibitory control over serotonergic projection neurons in the dorsal (DRD) and ventral (DRV) DRN subgroups, acting as serotonergic interneurons^11^,^56^. Thus, although the retinoraphe projection is strongest to the DRND/L, DRN-projecting RGCs may modulate the activity serotonergic neurons in other regions of the DRN either by direct input or indirectly via serotonergic and GABAergic interneurons thereby contributing to the serotonergic modulation of diverse processes.

Our understanding of the intra-DRN circuitry is far from complete and as new information is gathered our simple model of ON and OFF RGC input to the DRN will be refined. Recent technological advances such as optogenetic and *in vivo* fiber-optic recordings^57^ will open a multitude of opportunities for functional studies of the neural basis underlying retinal regulation of DRN functions.

## Additional Information

**How to cite this article**: Zhang, T. *et al*. ON and OFF retinal ganglion cells differentially regulate serotonergic and GABAergic activity in the dorsal raphe nucleus. *Sci. Rep.* **6**, 26060; doi: 10.1038/srep26060 (2016).

## Figures and Tables

**Table 1:** Quantification of c-Fos-expressing cells in the DRND/L and retina.

	Dark	Light	L-AP4-Light	Saline-Dark	Saline-Light	MNU-Dark	MNU-Light
c-Fos+	37.4 ± 3.6(n = 10)	201.6 ± 8.1(n = 10)	97.0 ± 8.6(n = 10)	44.8 ± 2.6(n = 8)	201.2 ± 8.2(n = 8)	566.5 ± 8.3(n = 10)	556.6 ± 6.8(n = 10)
c-Fos + /GABA+	35.6 ± 4.1(n = 5)	171.2 ± 16.9(n = 5)	28.8 ± 3.6(n = 5)	40.6 ± 3.7(n = 4)	165.7 ± 7.8(n = 4)	205.8 ± 7.3(n = 5)	199.6 ± 5.3(n = 5)
c-Fos + /TPH+	2.6 ± 0.9(n = 5)	33.0 ± 3.2(n = 5)	68.0 ± 5.6(n = 5)	2.4 ± 0.6(n = 4)	34.7 ± 3.8(n = 4)	331.2 ± 10.2(n = 5)	343.6 ± 12.5(n = 5)
c-Fos+ retinal cells/mm^2^	—	—	121.2 ± 16.4(n = 10)	14.2 ± 2.8(n = 5)	935.8 ± 23.5(n = 5)	192.5 ± 14.6(n = 5)	197.6 ± 10.7(n = 5)

All values = mean ± SEM; n = animals or retinas/group.
